# Admission electrolyte and osmotic pressure levels are associated with the incidence of contrast-associated acute kidney injury

**DOI:** 10.1038/s41598-022-08597-z

**Published:** 2022-03-18

**Authors:** Qingbo Lv, Duanbin Li, Yao Wang, Pengcheng Yu, Liding Zhao, Songzan Chen, Min Wang, Guosheng Fu, Wenbin Zhang

**Affiliations:** Key Laboratory of Cardiovascular Intervention and Regenerative Medicine of Zhejiang Province, Department of Cardiology, Sir Run Run Shaw Hospital, Zhejiang University School of Medicine, 3 East Qingchun Road, Hangzhou, Zhejiang Province People’s Republic of China

**Keywords:** Interventional cardiology, Risk factors

## Abstract

This retrospective study aimed to explore the relationships between electrolytes and osmotic pressure homeostasis with contrast-associated acute kidney injury (CA-AKI) risk in patients with percutaneous coronary intervention or coronary angiography. We totally enrolled 4386 hospitalized patients, who were categorized into five groups based on the predetermined cutoff values of electrolytes and osmotic pressure. CA-AKI was defined as an increase in serum creatine by 0.5 mg/dL (44.2 mol/L) or a 25% increase of the highest level post-operation compared to baseline. Multivariable logistic analysis was used to examine the association of CA-AKI incidence with electrolytes and osmotic pressure levels. Piecewise linear regression models and restricted cubic spline analysis were further utilized to determine the nonlinear relationship. The results showed U-shaped relationships between sodium, chloride, magnesium, and osmotic pressure levels and CA-AKI incidence. The lowest incidence was observed in the categories of 139–141.9 mmol/L, 107.0–109.9 mmol/L, 0.91–1.07 mmol/L, and 290.0–299.9 mOsm/kg, respectively. J-shaped associations were observed for potassium and phosphate levels and CA-AKI incidence, with the lowest incidence in the categories of 3.50–4.09 mmol/L and 0.96–1.28 mmol/L, respectively. A negative correlation was observed between calcium level and CA-AKI incidence, with the lowest CA-AKI risk in the category of ≥ 2.58 mmol/L. In conclusion, abnormally higher or lower sodium, chloride, magnesium, phosphate, and osmotic pressure levels on admission were associated with increased risks of CA-AKI. While for potassium and calcium, the status of hyperkalemia and hypocalcemia on admission showed more susceptibility for CA-AKI.

## Introduction

Iodized contrast medium (CM) is indispensable for interventional cardiac procedures. Iodized CM use is one of the most common causes of contrast-associated acute kidney injury (CA-AKI). It is the third leading cause of acute kidney injury (AKI) following renal hypoperfusion and drug-induced acute kidney injury during hospitalizations^1^. The incidence of CA-AKI is about 3.3% in the general population^2^, and patients with concomitant diseases such as chronic kidney disease, diabetes mellitus, and heart failure are at much higher risk^3,4^. Although CA-AKI is generally reversible, patients have a five times higher risk of in-hospital mortality than those without CA-AKI^5,6^. Understanding the CA-AKI mechanism and its risk factors is urgently needed to minimize the potential risks for patients undergoing percutaneous coronary intervention (PCI) and coronary angiography (CAG).

Electrolytes, such as sodium, chloride, potassium, calcium, magnesium, and phosphate, play crucial roles in regulating plasma osmolality, acid–base homeostasis, cellular metabolism, cellular membrane potentials of nerve and muscle cells^7^. Plasma osmotic pressure balance is critical to maintaining the fluid distribution across the cell membrane. Disturbance of electrolytes and osmotic pressure could induce significant clinical disorders, such as water intoxication, edema, neuromuscular dysfunction, and arrhythmia^8^. Electrolytes and osmotic pressure disturbances are prevalent in patients with cardiovascular disease (CVD)^9^. Abnormalities in these factors have been linked to poor prognosis in patients. Emerging evidence has proved that baseline electrolyte levels were independently associated with acute kidney injury for the hospitalized patients^10–14^. However, only one study described the association between the baseline electrolyte levels, specifically chloride levels, and CA-AKI^10^. Therefore, this is the first multicenter cohort study to explore the relationship between admission electrolyte and osmotic pressure levels with CA-AKI incidences.

## Results

### Baseline and procedural characteristics of all patients with or without CA-AKI

**Figure 1:**
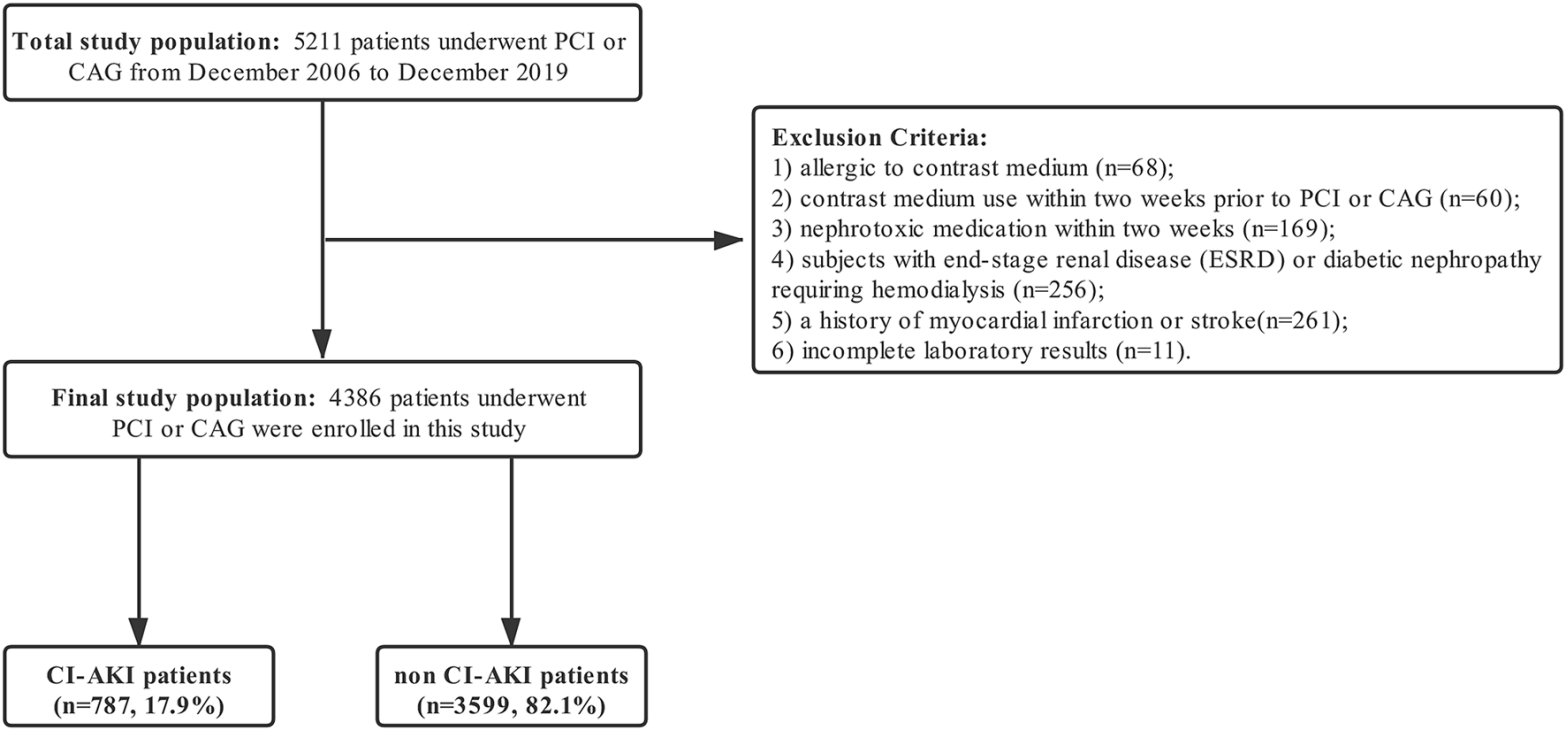
Flow chart of patients we enrolled in our study.

A total of 5211 patients were enrolled in this study. About 785 patients were excluded according to the criteria of enrollment. Finally, 4386 patients (age 67.10 ± 10.77 years) were enrolled for further analysis, of whom 787 (17.9%) were diagnosed with CA-AKI. The flow diagram of enrollment is shown in Fig. 1. Table 1 shows the baseline and angiographic characteristics of the patients based on their CA-AKI status. The patients in the CA-AKI group were older than those in the non-CA-AKI group (69.35 ± 10.65 vs. 66.61 ± 10.73 years, *P* < 0.001). Besides, they have a higher proportion of diabetes mellitus (DM) compared with patients in the non-CA-AKI group (28.2 vs. 22.2%, *P* = 0.004), as well as a higher HbA1c level observed in the CA-AKI group (6.70 ± 1.63 vs. 6.41 ± 1.33%, *P* < 0.001). They were also more likely presented with myocardial infarction (30.9 vs. 18.1%, *P* < 0.001) and had lower ejection fraction levels (56.57 ± 13.29 vs. 60.40 ± 12.89%, *P* < 0.001). The NT-proBNP levels were also significantly higher in the CA-AKI group (1522.00 (480.50–3634.00) *vs.* 513.00 (129.00–1681.00) pg/ml, *P* < 0.001). For the lipid profiles, the patients who developed CA-AKI had lower levels of total cholesterol (TC) (3.96 (3.13–4.61) *vs.* 3.96 (3.31–4.74) mmol/L, *P* = 0.006), high-density lipoprotein (HDL) (0.95 (0.76–1.12) *vs.* 0.98 (0.83–1.16) mmol/L, *P* < 0.001), very-low-density lipoprotein (VLDL) (0.58 (0.38–0.81) *vs*. 0.60 (0.41–0.88) mmol/L, *P*=0.002), and triglyceride (TG) (1.22 (0.89–1.63) *vs.* 1.28 (0.97–1.82) mmol/L, *P* < 0.001) than the non-CA-AKI patients. Patients in two groups had a similar distribution of low-density lipoprotein (LDL) (2.08 (1.54–2.72) *vs.* 2.08 (1.59–2.71) mmol/L, *P* = 0.485). Patients in the CA-AKI group have a slightly lower estimated glomerular filtration rate (eGFR) levels than those in the non-CA-AKI group (75.57 ± 28.04 *vs*. 79.37 ± 22.05 mL/min/1.73m^2^, *P* = 0.012). Potassium (4.05 ± 0.50 *vs.* 3.96 ± 0.40 mmol/L, *P* < 0.001), phosphate (1.16 ± 0.37 *vs.* 1.11 ± 0.23 mmol/L, *P* < 0.001), and osmotic pressure (292.53 ± 8.87 *vs.* 291.51 ± 6.53 mOsm/kg, *P* < 0.001) levels were higher in the CA-AKI group than the non-CA-AKI group. In contrast, the levels of sodium (138.80 ± 3.72 *vs.* 139.48 ± 2.99 mmol/L, *P* < 0.001), chloride (105.05 ± 4.43 *vs.* 105.67 ± 3.51 mmol/L, *P* < 0.001), calcium (2.20 ± 0.16 *vs.* 2.25 ± 0.14 mmol/L, *P* < 0.001), and magnesium (0.85 ± 0.14 *vs.* 0.86 ± 0.11 mmol/L, *P* = 0.010) were lower in the CA-AKI group compared with the non-CA-AKI group.

**Table 1:** The baseline and angiographic characteristics of the two groups.

	Overall	CA-AKI	Without CA-AKI	*P* value
	n = 4386	n = 787	n = 3599	
**Patient characteristics**				
Age, years	67.10 ± 10.77	69.35 ± 10.65	66.61 ± 10.73	< 0.001
Male, n (%)	2895 (66.0)	473 (60.1)	2422 (67.3)	< 0.001
BMI, %	24.45 ± 5.33	24.96 ± 5.25	24.35 ± 5.34	0.012
Hypertension, n (%)	2788 (63.6)	514 (65.3)	2274 (63.2)	0.279
Diabetes, n (%)	1058 (24.1)	222 (28.2)	836 (23.2)	0.004
Current smoker, n (%)	744 (17.0)	116 (14.7)	628 (17.4)	0.075
Current drinker, n (%)	674 (15.4)	98 (12.5)	576 (16.0)	0.014
Ejection fraction, %	59.70 ± 13.04	56.57 ± 13.29	60.40 ± 12.89	< 0.001
Coronary heart disease, n (%)	2841 (68.6)	524 (71.1)	2317 (68.0)	0.113
Myocardial infarction, n (%)	845 (20.4)	228 (30.9)	617 (18.1)	< 0.001
Proteinuria, n (%)	334 (8.1)	64 (8.7)	270 (8.0)	0.575
History of percutaneous coronary intervention, n (%)	230 (24.8)	39 (21.4)	191 (25.6)	0.287
History of myocardial infarction, n (%)	71 (7.7)	13 (7.2)	58 (7.9)	0.892
History of coronary artery bypass grafting, n (%)	15 (1.7)	4 (2.3)	11 (1.6)	0.752
**Laboratory data**				
Sodium, mmol/L	139.36 ± 3.14	138.80 ± 3.72	139.48 ± 2.99	< 0.001
Chloride, mmol/L	105.56 ± 3.70	105.05 ± 4.43	105.67 ± 3.51	< 0.001
Potassium, mmol/L	3.98 ± 0.42	4.05 ± 0.50	3.96 ± 0.40	< 0.001
Calcium, mmol/L	2.24 ± 0.15	2.20 ± 0.16	2.25 ± 0.14	< 0.001
Magnesium, mmol/L	0.86 ± 0.11	0.85 ± 0.14	0.86 ± 0.11	0.010
Phosphate, mmol/L	1.12 ± 0.26	1.16 ± 0.37	1.11 ± 0.23	< 0.001
Osmotic pressure, mOsm/kg	291.69 ± 7.02	292.53 ± 8.87	291.51 ± 6.53	< 0.001
Pre-operative creatinine, μmol/L	76.00 (64.00–94.00)	73.00 (60.00–100.00)	76.00 (65.00–93.00)	0.090
Post-operative creatinine, μmol/L	81.00 (67.00–103.00)	112.00 (87.00–164.50)	77.00 (65.00–93.50)	< 0.001
Percentage of creatinine elevation, %	5.20 (−3.90–18.20)	43.20 (32.40–68.10)	1.90 (−5.70–10.10)	< 0.001
eGFR, mL/min/1.73m^2^	78.68 ± 23.28	75.57 ± 28.04	79.37 ± 22.05	0.012
NT-proBNP, pg/ml	641.00 (158.00–2026.75)	1522.00 (480.50–3634.00)	513.00 (129.00–1681.00)	< 0.001
HbA1c, %	6.45 ± 1.38	6.70 ± 1.63	6.41 ± 1.33	< 0.001
Uric acid, μmol/L	364.00 (297.00–432.00)	364.00 (288.50–438.00)	364.00 (299.00–430.00)	0.504
Total bilirubin, μmol/L	13.20 (9.60–18.50)	13.40 (9.30–19.70)	13.20 (9.70–18.20)	0.272
Total cholesterol, mmol/L	3.96 (3.27–4.72)	3.96 (3.13–4.61)	3.96 (3.31–4.74)	0.006
Low-density lipoprotein, mmol/L	2.08 (1.58–2.71)	2.08 (1.54–2.72)	2.08 (1.59–2.71)	0.485
High-density lipoprotein, mmol/L	0.98 (0.82–1.16)	0.95 (0.76–1.12)	0.98 (0.83–1.16)	< 0.001
Very-low-density lipoprotein, mmol/L	0.60 (0.40–0.87)	0.58 (0.38–0.81)	0.60 (0.41–0.88)	0.002
Triglyceride, mmol/L	1.28 (0.95–1.79)	1.22 (0.89–1.63)	1.28 (0.97–1.82)	< 0.001
White blood cell, × 10^9^/L	7.27 ± 2.98	8.25 ± 3.87	7.05 ± 2.71	< 0.001
Hemoglobin, g/L	130.55 ± 19.57	123.06 ± 20.97	131.62 ± 19.14	< 0.001
Platelet, × 10^9^/L	184.91 ± 67.79	187.74 ± 77.35	184.29 ± 65.50	0.196
C-reactive protein, mg/L	2.30 (0.90–8.00)	4.40 (1.45–16.00)	2.00 (0.80–6.60)	< 0.001
Perioperative mean heart rate, per min	74.34 ± 43.15	76.75 ± 18.95	73.81 ± 46.79	0.084
Perioperative mean SBP, mmHg	123.21 ± 14.42	120.62 ± 15.46	123.77 ± 14.12	< 0.001
Perioperative mean DBP, mmHg	69.78 ± 8.82	67.08 ± 8.41	70.37 ± 8.79	< 0.001
**Percutaneous coronary intervention characteristics**				
Predilation, n (%)	1231 (87.2)	228 (87.0)	1003 (87.3)	0.987
Postdilation, n (%)	1634 (96.0)	289 (94.4)	1345 (96.3)	0.168
FFR/IVUS/OCT, n (%)	288 (22.1)	29 (13.4)	259 (23.8)	0.001
Chronic total occlusion, n (%)	207 (16.2)	38 (16.7)	169 (16.1)	0.880
Multivessel lesion, n (%)	79 (6.7)	14 (6.9)	65 (6.6)	1.000
Target imaging vessel, n (%)				
LM	137 (11.0)	24 (11.5)	113 (10.8)	0.882
LAD	959 (63.9)	186 (70.2)	773 (62.5)	0.023
LCX	358 (26.4)	60 (26.1)	298 (26.5)	0.971
RCA	493 (36.1)	86 (36.8)	407 (36.0)	0.890
**Type of contrast agent, n (%)**				0.001
Ioversol	202 (4.6)	16 (2.0)	186 (5.2)	
Iopamidol	2783 (63.8)	521 (66.4)	2262 (63.2)	
Iodixanol	1380 (31.6)	248 (31.6)	1132 (31.6)	
Isotonic contrast agent, n (%)	1380 (31.6)	248 (31.6)	1132 (31.6)	1.000
**Pre-admission medication, n (%)**				
Statin	3655 (83.3)	603 (76.6)	3052 (84.8)	< 0.001
Aspirin	3632 (82.8)	571 (72.6)	3061 (85.1)	< 0.001
Clopidogrel	3093 (70.5)	438 (55.7)	2655 (73.8)	< 0.001
Ticagrelor	759 (17.3)	167 (21.2)	592 (16.4)	0.002
Furosemide injection	660 (15.0)	239 (30.4)	421 (11.7)	< 0.001
Torasemide injection	133 (3.0)	67 (8.5)	66 (1.8)	< 0.001
Dopamine	1231 (28.1)	302 (38.4)	929 (25.8)	< 0.001

*BMI* body mass index, *eGFR* estimated glomerular filtration rate, *NT-proBNP* N- terminal pro-B-type natriuretic peptide, *LM* left main, *LAD* left anterior descending, *LCX* circumflex, *RCA* right coronary artery.

### The distributions of CA-AKI incidence among patients with different categories of the electrolyte and osmotic pressure

**Figure 2:**
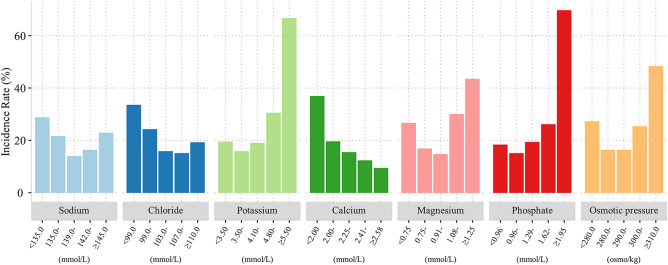
The incidence of CA-AKI in each category of electrolytes and osmotic pressure. All patients were divided into fifth according to the pre-defined cut-off points.

To explore the relationships of baseline electrolyte and osmotic pressure levels with CA-AKI incidence, we first divided all patients into five groups based on the predetermined values of each kind of electrolyte and osmotic pressure. Figure 2 shows the incidence of CA-AKI in each group. For sodium and chloride, the highest CA-AKI incidences were both observed in the first group. The lowest CA-AKI incidences were observed in the third and fourth groups for sodium and chloride, respectively. In contrast, the fifth groups of potassium, magnesium, phosphate, and osmotic pressure showed the highest rates of CA-AKI, which meant that abnormally high levels of these factors have the most chances for the presence of CA-AKI. Finally, a negative correlation between calcium levels and CA-AKI incidence was observed, showing that the incidence of CA-AKI decreased with an increase in calcium levels.

### The hidden correlations among the distributions of electrolytes and osmotic pressure

**Figure 3:**
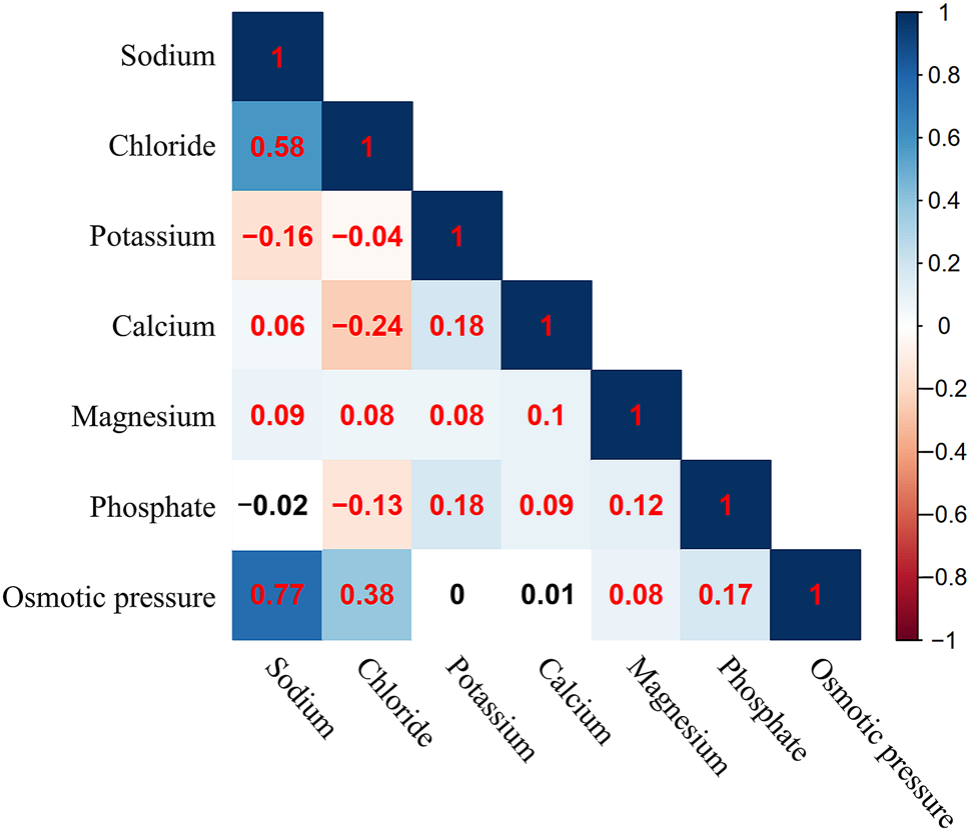
The correlation matrix among the levels of potassium, sodium, chloride, calcium, magnesium, phosphate, and osmotic pressure. The value of correlation coefficient between any of the two variables was marked in the Figure. The correlation coefficients in red indicated that the correlation between two variables is statistically significant (*P* < 0.05), while the correlation coefficients in black indicated no statistical significance.

Since we studied seven different independent variables simultaneously, a correlation matrix was first conducted to detect hidden patterns among these seven variables. As shown in Fig. 3, a significantly positive correlation was observed between the levels of sodium and osmotic pressure (correlation coefficient = 0.77), so did the association between sodium and chloride levels (correlation coefficient = 0.58). These two positive relationships are in accordance with previous well-known findings^15^. Other correlation coefficients of each pair were all shown in Fig. 3. The correlation coefficients listed were all statistically significant except for the ones for sodium and phosphate, potassium and osmotic pressure, calcium and osmotic pressure.

### The association between the distribution of electrolyte and osmotic pressure and risk of CA-AKI

Since we have found the segmentation characteristics of CA-AKI incidence among different electrolytes and osmotic pressure, we further performed logistic regression analysis for each variable to determine whether different concentrations acted as influential factors for CA-AKI. A multivariate logistic model was utilized by adjusting for potential confounders. We used three different models to calculate the odds ratios, 95% CI, and *P*-value for trend, as shown in Table 2. The lowest CA-AKI incidence category of each variable was set as the reference, except for calcium. The third category of calcium was selected as the reference group.

**Table 2:** Odds ratio (95% CI) of CI-AKI according to the categories of electrolytes and osmotic pressure.

	Ratio of CI-AKI (%)	Model 1*	*P* for trend	Model 2†	*P* for trend	Model 3‡	*P* for trend
		Odds ratio (95% CI)		Odds ratio (95% CI)		Odds ratio (95% CI)	
**Sodium**							
< 135.0 mmol/L	81/281 (28.8)	2.313 (1.726 to 3.101)	< 0.001	2.359 (1.755 to 3.172)	< 0.001	1.897 (1.395 to 2.581)	< 0.001
135.0–138.9 mmol/L	276/1269 (21.7)	1.668 (1.383 to 2.011)		1.721 (1.424 to 2.08)		1.621 (1.334 to 1.97)	
139.0–141.9 mmol/L	264/1881 (14.0)	1 (reference)		1 (reference)		1 (reference)	
142.0–144.9 mmol/L	133/811 (16.4)	1.184 (0.943 to 1.488)	0.012	1.165 (0.926 to 1.465)	0.021	1.172 (0.926 to 1.484)	0.054
≥ 145.0 mmol/L	33/144 (22.9)	1.811 (1.198 to 2.738)		1.757 (1.16 to 2.661)		1.636 (1.063 to 2.518)	
**Chloride**							
< 99.0 mmol/L	57/170 (33.5%)	2.577 (1.799 to 3.691)	< 0.001	2.555 (1.78 to 3.667)	< 0.001	1.861 (1.275 to 2.715)	0.001
99.0–102.9 mmol/L	155/639 (24.3%)	1.743 (1.373 to 2.213)		1.756 (1.381 to 2.234)		1.423 (1.107 to 1.828)	
103.0–106.9 mmol/L	290/1823 (15.9%)	1.072 (0.878 to 1.307)		1.072 (0.878 to 1.309)	0.495	1.03 (0.841 to 1.263)	
107.0–109.9 mmol/L	193/1275 (15.1%)	1 (reference)		1 (reference)		1 (reference)	
≥ 110.0 mmol/L	92/479 (19.2%)	1.331 (1.01 to 1.753)	0.042	1.35 (1.024 to 1.781)	0.034	1.345 (1.01 to 1.792)	0.043
**Potassium**							
< 3.50 mmol/L	91/467 (19.5)	1.2 (0.929 to 1.552)	0.163	1.186 (0.916 to 1.536)	0.196	1.157 (0.886 to 1.511)	0.288
3.50–4.09 mmol/L	377/2364 (15.9)	1 (reference)		1 (reference)		1 (reference)	
4.10–4.79 mmol/L	264/1393 (19.0)	1.19 (0.998 to 1.418)	< 0.001	1.199 (1.005 to 1.431)	< 0.001	1.203 (1.004 to 1.442)	< 0.001
4.80–5.49 mmol/L	45/147 (30.6)	2.176 (1.492 to 3.173)		2.177 (1.491 to 3.18)		1.944 (1.31 to 2.887)	
≥ 5.50 mmol/L	10/15 (66.7)	10.114 (3.396 to 0.122)		10.938 (3.641 to 32.857)		9.386 (2.983 to 29.536)	
**Calcium**							
< 2.00 mmol/L	74/200 (37.0)	2.949 (2.142 to 4.059)	< 0.001	3.009 (2.178 to 4.155)	< 0.001	2.613 (1.87 to 3.652)	< 0.001
2.00–2.24 mmol/L	397/2030 (19.6)	1.261 (1.06 to 1.501)		1.303 (1.093 to 1.553)		1.221 (1.02 to 1.462)	
2.25–2.40 mmol/L	255/1647 (15.5)	1 (reference)		1 (reference)		1 (reference)	
2.41–2.57 mmol/L	55/446 (12.3)	0.821 (0.599 to 1.125)	0.212	0.789 (0.574 to 1.084)	0.115	0.841 (0.608 to 1.164)	0.330
≥ 2.58 mmol/L	6/63 (9.5)	0.556 (0.236 to 1.308)		0.499 (0.211 to 1.183)		0.604 (0.253 to 1.442)	
**Magnesium**							
< 0.75 mmol/L	151/566 (26.7)	1.968 (1.519 to 2.55)	< 0.001	1.93 (1.488 to 2.504)	< 0.001	1.748 (1.335 to 2.288)	< 0.001
0.75–0.90 mmol/L	422/2504 (16.9)	1.143 (0.936 to 1.397)		1.145 (0.936 to 1.399)		1.086 (0.884 to 1.334)	
0.91–1.07 mmol/L	174/1197 (14.5)	1 (reference)		1 (reference)		1 (reference)	
1.08–1.24 mmol/L	27/89 (30.3)	2.341 (1.368 to 4.005)	< 0.001	2.382 (1.386 to 4.094)	< 0.001	2.328 (1.312 to 4.132)	< 0.001
≥ 1.25 mmol/L	13/30 (43.3)	3.92 (1.669 to 9.206)		3.966 (1.671 to 9.415)		2.814 (1.12 to 7.07)	
**Phosphate**							
< 0.96 mmol/L	197/1047 (18.8)	1.232 (0.996 to 1.524)	0.055	1.345 (1.082 to 1.673)	0.008	1.356 (1.083 to 1.698)	0.008
0.96–1.28 mmol/L	383/2468 (15.5)	1 (reference)		1 (reference)		1 (reference)	
1.29–1.61 mmol/L	133/699 (19.0)	1.39 (1.095 to 1.765)	< 0.001	1.317 (1.034 to 1.677)	< 0.001	1.318 (1.028 to 1.689)	< 0.001
1.62–1.94 mmol/L	28/105 (26.7)	1.843 (1.034 to 3.286)		1.838 (1.029 to 3.286)		1.443 (0.779 to 2.673)	
≥ 1.95 mmol/L	46/67 (68.7)	12.318 (5.652 to 6.846)		12.31 (5.627 to 26.932)		14.348 (6.162 to 33.41)	
**Osmotic pressure**							
< 280.0 mOsm/kg	45/165 (27.3)	1.752 (1.218 to 2.519)	0.008	1.838 (1.275 to 2.649)	0.005	1.528 (1.045 to 2.234)	0.090
280.0–289.9 mOsm/kg	249/1519 (16.4)	0.993 (0.832 to 1.185)		1.058 (0.883 to 1.267)		1.029 (0.856 to 1.237)	
290.0–299.9 mOsm/kg	376/2294 (16.4)	1 (reference)		1 (reference)		1 (reference)	
300.0–309.9 mOsm/kg	89/350 (25.4)	1.681 (1.277 to 2.213)	< 0.001	1.65 (1.252 to 2.176)	< 0.001	1.65 (1.252 to 2.176)	< 0.001
≥ 310.0 mOsm/kg	28/58 (48.3)	4.811 (2.787 to 8.305)		4.69 (2.715 to 8.104)		4.69 (2.715 to 8.104)	

*Model 1: Adjusted for age (< 65 years or ≥ 65 years) and eGFR (< 30, 30–59, 60–89, or ≥ 90 mL/min/1.73m^2^).

^**†**^Model 2: Adjusted for age (< 65 years or ≥ 65 years) and eGFR (< 30, 30–59, 60–89, or ≥ 90 mL/min/1.73m^2^), gender (male or female), diabetes (yes or no), hypertension (yes or no), current smoker (yes or no),current drinker (yes or no).

^**‡**^Model 3: Additionlly adjusted for type of contrast agent (ioversol, iopamidol, or iodixanol), pre-admission of furosemide injection (yes or no), pre-admission of dopamine (yes or no).

**Figure 4:**
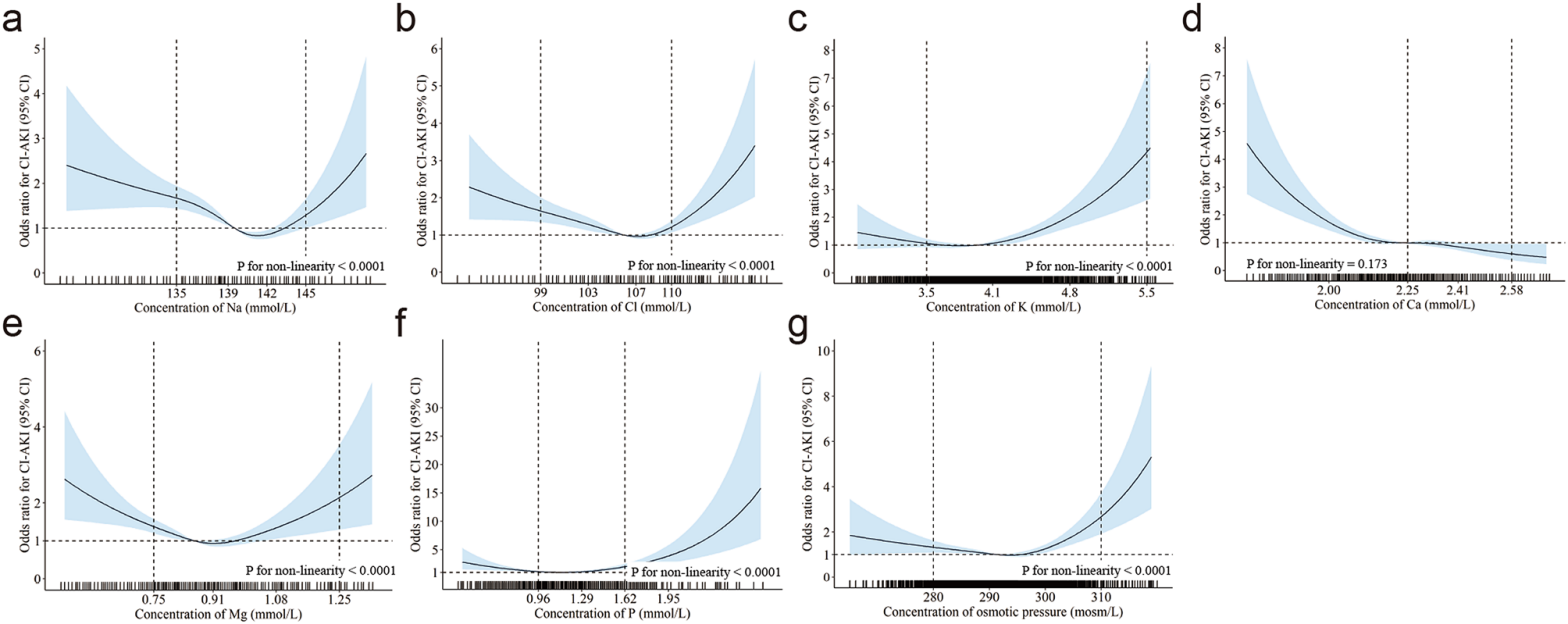
Piecewise linear curve fitting by restricted cubic spline models for the association of sodium (**a**), chloride (**b**), potassium (**c**), calcium (**d**), magnesium (**e**), phosphate (**f**), and osmotic pressure (**g**) distributions with CA-AKI incidence. Models adjusted for age(< 65 or ≥ 65 years) and eGFR (< 30, 30–59, 60–89, or ≥ 90 mL/min/1.73 m^2^), gender (male or female), diabetes (yes or no), hypertension (yes or no), current smoker (yes or no), current drinker (yes or no), type of contrast agent (ioversol, iopamidol, or iodixanol), pre-admission of furosemide injection (yes or no), pre-admission of dopamine (yes or no). Odds ratios are indicated by solid lines and 95% confidence intervals by shaded areas. The lowest value of CA-AKI incidence was selected as the reference point for sodium, chloride, potassium, magnesium, phosphate, osmotic pressure. The third category was selected as the reference point for calcium.

To flexibly model and visualize the relation of these electrolyte and osmotic pressure levels with CA-AKI incidence, we performed restricted cubic splines to visualize the data of Model 3 (Fig. 4). The levels of sodium, chloride, magnesium, and osmotic pressure showed strong U-shaped associations with CA-AKI incidences. Specifically, as shown in Fig. 4, a substantial reduction of risk for CA-AKI was observed with the elevation of concentrations, reaching the bottom in the categories of 139.0–141.9 mmol/L, 107.0–109.9 mmol/L, 0.91–1.07 mmol/L, and 290.0–299.9 mOsm/kg for sodium, chloride, magnesium, and osmotic pressure, respectively (*P* for trend were < 0.001, 0.001, < 0.001, and 0.090, respectively). Then the risk increased with the rise of concentrations (*P* for trend were 0.054, 0.043, < 0.001, and < 0.001, respectively). For potassium and phosphate, the lowest CA-AKI incidence was observed in the second category, showing overall J-shape association patterns. For potassium, the risk for CA-AKI was relatively flat until the category of 3.50–4.19 mmol/L (*P* for trend was 0.288), and then the risk started to rise rapidly afterward (*P* for trend was < 0.001). For phosphate, levels lower or higher than the category of 0.96–1.28 mmol/L increased the risk of CA-AKI significantly (*P* for trend were 0.008 and < 0.001). Uniquely, a negative correlation of calcium level with CA-AKI incidence was observed when the concentration was lower than the category of 2.25–2.40 mmol/L (*P* for trend was < 0.001). The curve then became relatively flat, indicating that the risk of CA-AKI did not significantly increase with the rise of calcium concentration when it was more than 2.40 mmol/L (*P* for trend was 0.330). The specific odds ratio values of each category compared with the reference group are presented in Table 2.

### Subgroup analysis of age, dose of contrast medium, eGFR, DM, and HbA1c for the analysis of the CA-AKI incidence

The serum creatinine (Cr) level is a critical reference parameter for the kidney injury evaluation and diagnosis of CA-AKI. However, it can be affected by several factors such as age, the dose of contrast agent, eGFR, and DM. So we conducted subgroup analysis stratified by these factors and presented them as restricted cubic splines in Supplemental Figs. 1–4. It shows the unique variation characteristics for the relationship between electrolytes and osmotic pressure levels and CA-AKI risks among different subgroups. The subgroup analysis of age showed that the trends of these electrolytes and osmotic pressure with CA-AKI were similar among patients ≥ 70 years and < 70 years (Supplemental Fig. 1). On the other hand, hypernatremia and hyperchloremia could slightly increase the CA-AKI risk among patients ≥ 70 years than the patients < 70 years. When the contrast agent was ≥ 100 ml, the hyponatremia, hyperchloremia, and hypomagnesemia showed higher risks of CA-AKI than the patients receiving contrast media < 100 ml (Supplemental Fig. 2). For patients with eGFR < 90 ml/min/1.73m^2^, a higher potassium level significantly increased the risk of CA-AKI. In addition, hypomagnesemia and hyperphosphatemia showed unique higher risks of CA-AKI compared with patients with eGFR < 90 ml/min/1.73m^2^ (Supplemental Fig. 3). Furthermore, higher risks for CA-AKI incidence among patients with DM were observed in the lower sodium, chloride, and osmotic pressure categories than non-DM patients (Supplemental Fig. 4). Lastly, we performed the subgroup analysis for all enrolled patients according to their HbA1c values to further explore the influence of glycaemic control on the CI-AKI. Similar to the subgroup analysis of DM, for patients with HbA1c ≥ 6.5%, hyponatremia, hyperchloremia, hypocalcemia, hypophosphatemia, and hypoosmolaemia showed higher risks for CA-AKI than those with HbA1c < 6.5% (Supplemental Fig. 5).

## Discussion

Previous studies have revealed that AKI could induce abnormal electrolyte levels since the kidney is the primary organ for maintaining the homeostasis of electrolytes^16,17^. However, it is unclear whether the distribution of electrolytes could predict CA-AKI. Up to our knowledge, this is the first study focusing on the associations between admission electrolyte and osmotic pressure levels and risk of CA-AKI. The main finding of this study is that CA-AKI incidence showed different patterns of associations with electrolytes and osmotic pressure homeostasis; U-shaped associations with serum sodium, chloride, magnesium, and osmotic pressure, J-shaped association with potassium and phosphate, and an inverse relationship with calcium levels.

Several studies have reported the significance of these electrolyte and osmotic pressure levels on the incidence of acute kidney injury. In this study, we firstly performed a correlation matrix to explore the underlying relations among these variables. Although most correlations between two variables were statistically significant, the low correlation coefficients, except for the one of sodium and osmotic pressure and the one of sodium and chloride, indicated that the different electrolytes and osmotic pressure affected the incidence of CA-AKI in their unique roles. Since prior studies mainly focused on reporting the links between these electrolytes and AKI, but not with CA-AKI, we can only compare our findings with previous studies of AKI. Sodium is the primary electrolyte responsible for osmolarity^15^, evidenced by the highest correlation coefficient in the correlation matrix of our research. The risk patterns of sodium and osmotic pressure were also similar, both as U-shapes. Similar to our results, a recent study by Chen et al.^18^ revealed that hypernatremia and hyponatremia were both associated with increased risk for AKI. The mechanism for hypernatremia inducing AKI might be due to its effect on intravascular dehydration and vasoconstriction by tubuloglomerular feedback^19,20^.

Chloride is another important contributor to osmotic pressure, with a normal range of 96.0–114.0 mmol/L. The extracellular chloride levels are proved to be crucial for the constriction of the afferent arteriole, mesangial cells, and juxtaglomerular cells^21^. Oh et al*.*^10^ reported that both patients with hypochloremia and hyperchloremia have higher CA-AKI rates than patients with normal chloride levels. This result is in agreement with our multivariable logistic regression analysis and subgroup analysis. The impact of chloride on AKI might be due to the regulating role of chloride for tubuloglomerular feedback and renin secretion^21^.

The imbalance of potassium homeostasis is prevalent in patients with impaired renal function. Park et al*.*^11^ found serum potassium at baseline over 4.0 mmol/L was a significant risk factor for in-hospital acute kidney injury, which was similar to our results. We found that hypokalemia did not significantly increase the risk for CA-AKI. However, higher baseline potassium levels over 4.1 mmol/L significantly increased the risk for CA-AKI compared with the reference group. This result mainly emphasizes the risks of hyperkalemia for CA-AKI. It might be that hyperkalemia is regarded as a sign of renal dysfunction and some comorbid diseases.

Electrolytes like calcium, magnesium, and phosphate are essential minerals for many physiological functions of the body, including maintaining normal cardiac function, neuronal activity, and bone health^22^. Calcium is a critical mineral required for several physiologic functions, such as intracellular messenger transduction and hormonal activity^12^. Also, calcium affects the vascular tone of the kidney. The imbalance between renal vasoconstriction and vasodilatation due to the abnormality of calcium finally induces AKI^23^. Thongprayoon et al*.*^12^ observed a U-shaped association between admission serum ionized calcium and in-hospital AKI. Both hypocalcemia and hypercalcemia on admission were associated with an elevated risk of in-hospital AKI. However, our study found a negative linear association between serum calcium levels and CA-AKI incidence. The highest CA-AKI incidence was presented in the categories of serum calcium < 2.00 mmol/L. Compared with the reference group, higher calcium levels did not significantly increase the risk, which might be due to the limited patients in the groups of hypercalcemia.

Magnesium is an essential catalyst in multiple cellular function^24^. It can counteract vasoconstriction by endogenous catecholamines and potentiate the action of endogenous vasodilators^25^. In a study by Shen et al.^13^, they found that low serum magnesium was an independent risk factor for AKI in patients with malignancy, while Cheungpasitporn et al*.*^26^ revealed that both admission hypomagnesemia and hypermagnesemia increased the risk for AKI. A U-shaped association between magnesium levels and CA-AKI incidence was also found in our study. The underlying mechanism for hypomagnesemia and hypermagnesemia causing AKI is that they dysregulate vascular tonicity homeostasis by overstimulated vasoconstriction and vasodilation effects, respectively^26^.

The kidney is the primary organ regulating phosphate levels. In a study investigating the predictive effect of serum phosphate for AKI in pediatric cardiac surgical patients, serum phosphate level was a reliable predictor of AKI with an AUC of 0.86^27^. Besides, hyperphosphatemia was proved associated with increased AKI incidence and higher risk for end-stage renal disease (ESRD)^28^. The potential mechanisms may involve the calcification of smooth muscle cells and disrupted endothelial function by the hyperphosphateimia^29^. In our study, both hypophosphatemia and hyperphosphatemia showed a significantly increased risk of CA-AKI. The overall relationship showed a J-shaped pattern. It provides new evidence on the risks of hypophosphatemia for CA-AKI.

Diabetes is a critical risk factor both for the CA-AKI and imbalance of electrolytes^30,31^. So we further performed subgroup analyses according to the presence of DM or not and baseline HbA1c levels. The results showed that intensive attention on hyponatremia, hypochloremia, and hypoosmolaemia should be paid to the DM patients to prevent CA-AKI. In addition, for the patients with HbA1c ≥ 6.5%, hyponatremia, hypermagnesemia, hypoosmolaemia, and hypoosmolaemia showed unique higher risks of CA-AKI compared with those patients with HbA1c < 6.5%.

All these studies combined with ours show the significant association between the electrolyte and osmotic pressure homeostasis and CA-AKI. The effective therapy to prevent CA-AKI is hydration, an inexpensive and beneficial treatment^32^. Such strategies lie in the oral or intravenous infusion with isotonic saline or sodium bicarbonate^33,34^. By adding the fluid, hydration increases the renal blood flow and decreases CM concentration in renal tubules, thus effectively reducing the toxicity of CM^35^.

Although the effectiveness of hydration therapy has been well recognized, the agents, timing, dose, and velocity of optimal hydration strategy remain inconclusive^36–38^. Debates mainly focused on the choice of intravenous normal saline infusion or intravenous sodium bicarbonate infusion. Intravenous hydration of sodium chloride is the most commonly used strategy to prevent CA-AKI in clinical practice, including our study. However, some researchers proposed that sodium chloride is a chloride-rich solution. According to our findings, hyperchloremia is a significant risk factor for CA-AKI. Hence, intravenous sodium bicarbonate infusion has been proposed. Another theoretical benefit of sodium bicarbonate is that alkalization with bicarbonate perfusion could decrease the formation of reactive oxygen species in the renal medulla^39^. However, whether an intravenous infusion of sodium bicarbonate is superior to sodium chloride in preventing CA-AKI still needs further large sample studies as proof^40–43^.

There are two main applications for this study. First, our findings remind the clinicians to pay close attention to the patients with electrolyte or osmotic pressure imbalance before PCI or CAG. Close monitoring and timely correction of the electrolyte and osmotic pressure imbalance are indispensable and beneficial to minimize the incidence of CA-AKI. We also provided optimal intervals of the lowest risk of AKI for each type of electrolyte and osmotic pressure. Second, the associations between sodium, chloride, and osmotic pressure and CA-AKI incidence revealed that the middle interval of the normal range was the lowest for the CA-AKI risk, indicating that osmotic normal saline hydration therapy might be the optimal strategy to prevent CA-AKI in normal patients. Applications of intravenous hydration strategies with isotonic or half iso-osmolar normal saline are left to the discretion of clinicians according to the baseline sodium, chloride, and osmotic pressure levels of each individual.

There were several limitations to this study. First, this is a retrospective observational study, and hence inherent bias was unavoidable. Second, our study did not provide data about the previous treatment that could influence electrolyte levels, such as diuretics, nor did we bring these confounding factors into our analytical model. Third, we only assessed data of total calcium concentration rather than ionized calcium in this study. It would be more scientific and reasonable to determine the relation between ionized calcium levels and CA-AKI. Besides, since the categories were predetermined according to the normal range, the number of patients across categories was unequal. One major consequence was that the number of patients in the first and fifth categories was limited. Although the numbers were sufficient to provide a trend of the odds ratio, they were insufficient to reflect a more solid odds ratio of this category. Lastly, we only have information on single serum electrolyte and osmotic pressure levels on admission. Therefore, we could not determine the time-course of changes of these levels that might influence the risk of CA-AKI.

In conclusion, admission electrolyte and osmotic pressure levels are associated with the incidence of CA-AKI in patients who underwent PCI or CAG. Abnormally higher or lower sodium, chloride, magnesium, phosphate, and osmotic pressure levels on admission were associated with increased risks of CA-AKI. While for potassium and calcium, the status of hyperkalemia and hypocalcemia on admission showed more susceptibility for CA-AKI.

## Methods

### Study population and design

It is a multicenter retrospective observational study. Consecutive hospitalized patients, who underwent PCI or CAG procedure at Sir Run Run Shaw Hospital and its medical consortium hospitals from December 2006 to December 2019, were enrolled in this study. For this study, our exclusion criteria included: (1) allergic to contrast medium; (2) contrast medium use within two weeks prior to PCI or CAG; (3) nephrotoxic medication within two weeks, including cisplatin, acyclovir, aminoglycoside antibiotics, and non-steroidal anti-inflammatory drug (NSAIDs) et al.; (4) subjects with end-stage renal disease (ESRD) or diabetic nephropathy requiring hemodialysis; (5) a history of myocardial infarction or stroke; (6) incomplete laboratory results. Patients were then divided into two groups according to their CA-AKI status. The study was performed following the criteria set by the Declaration of Helsinki involving experimenting on human subjects and approved by the Institutional Ethics Committee of Sir Run Run Hospital (20200803-34). Informed consent was obtained from all participants. The present study followed the Strengthening the Reporting of Observational Studies in Epidemiology (STROBE) reporting guideline.

### Study procedures and definitions

CAG or PCI procedures were performed by experienced interventional cardiologists according to the current standard guidelines^44^. The amount of contrast medium used in the procedure was left to the discretion of the cardiologists according to the progress of the operation. Before the procedures, all patients received 500 ml 0.9% normal saline (100 ml/h) intravenously for hydration therapy. Serum creatinine levels of patients were measured on admission and monitored at least three times within 72 h post-procedure. The highest level of serum creatinine after the procedure was recorded and used for comparison with the one on admission. CA-AKI was defined as an increase in serum creatine by 0.5 mg/dL (44.2 mol/L) or a 25% increase of the highest level within 72 h after the procedure compared to baseline^45^.

### Data collection

The blood samples of the enrolled patients were collected on admission and post-procedural for blood chemistry tests. The serum electrolyte, osmotic pressure, and creatinine levels were measured by the clinical chemistry analyzer (Beckman Coulter, California, USA). The demographic information of each patient was recorded, including age, gender, body mass index (BMI), comorbidities, smoking status, alcohol drinking status, the history of cardiovascular and renal diseases, and the history of PCI and coronary artery bypass grafting (CABG). The features of coronary lesions and procedural characteristics during the CAG or PCI, and the concomitant medications before admission, were also recorded. Proteinuria was defined as the urinary protein detected higher than 150 mg/d. eGFR was calculated by the value of creatine and patients’ age and weight. The formula is as follow: eGFR = (140-age) × weight / (72 × Cr) for male and eGFR = 0.85 × (140-age) × weight / (72 × Cr) for female.

### Statistical analysis

The categorical variables were expressed as numbers and percentages, while continuous variables were expressed as mean ± standard deviation (SD) or median and interquartile range. Categorical variables between groups were compared using chi-square tests, while continuous variables were compared with non-parametric Mann–Whitney U test.

The correlation matrix plot was used to evaluate the correlations among different electrolytes and osmotic pressure. Considering the normal ranges of electrolyte and osmotic pressure and characteristics of data distribution, patients were categorized into five groups according to predetermined cutoff points: sodium (< 135.0, 135.0–138.9, 139.0–141.9, 142.0–144.9, and ≥ 145.0 mmol/L), chloride (< 99.0, 99.0–102.9, 103.0–106.9, 107.0–109.9, and ≥ 110.0 mmol/L), potassium (< 3.50, 3.50–4.09, 4.10–4.79, 4.80–5.49, and ≥ 5.50 mmol/L), calcium (< 2.00, 2.00–2.24, 2.25–2.40, 2.41–2.57, and ≥ 2.58 mmol/L), magnesium (< 0.75, 0.75–0.90, 0.91–1.07, 1.08–1.24, and ≥ 1.25 mmol/L), phosphate (< 0.96, 0.97–1.28, 1.29–1.61, 1.62–1.94, and ≥ 1.95 mmol/L), and osmotic pressure (< 280.0, 280.0–289.9, 290.0–299.9, 300.0–309.9, and ≥ 310.0 mOsm/kg). Multivariate logistic regression analyses were used to examine the associations of pre-procedural electrolyte and osmotic pressure levels with CA-AKI incidence. The adjusted variables were age, eGFR, gender, diabetes, hypertension, smoking, drinking, type of contrast agent, pre-admission of furosemide injection, and pre-admission of dopamine. Restricted cubic spline analysis was used to fit and visualize these associations with 4 knots flexibly. Additionally, the piecewise *P*-value for the trend (*P* for trend) was calculated by treating the categories as continuous variables. Underlying non-linearity association was estimated by using a likelihood ratio test, and *P*-value for non-linearity was shown. Exploratory analyses were performed in subgroups stratified by age, eGFR, contrast volume, DM, and HbA1c levels. The results of subgroup analyses were all adjusted with the same clinically relevant variables above.

A two-tailed *P* < 0.05 was considered statistically significant. The statistical analyses were performed by SPSS (Version 18.0 SPSS Inc., Chicago, IL, USA) and R (version 3.5, The R Foundation for Statistical Computing, Vienna, Austria).

### Ethics approval

The study was conducted according to the Declaration of Helsinki, and was approved by the ethics committee of Sir Run Run Shaw Hospital (NO. 20200803-34).

## Supplementary Information

Supplementary Information 1.

Supplementary Information 2.

Supplementary Information 3.

Supplementary Information 4.

Supplementary Information 5.

Supplementary Information 6.

## Data Availability

Data are available from the corresponding author on reasonable request.
