# Supplementary figures and images for: Admission electrolyte and osmotic pressure levels are associated with the incidence of contrast-associated acute kidney injury
Source: Sci Rep. 2022 Mar 18;12:4714. doi: 10.1038/s41598-022-08597-z (PMC8933572; doi:10.1038/s41598-022-08597-z)

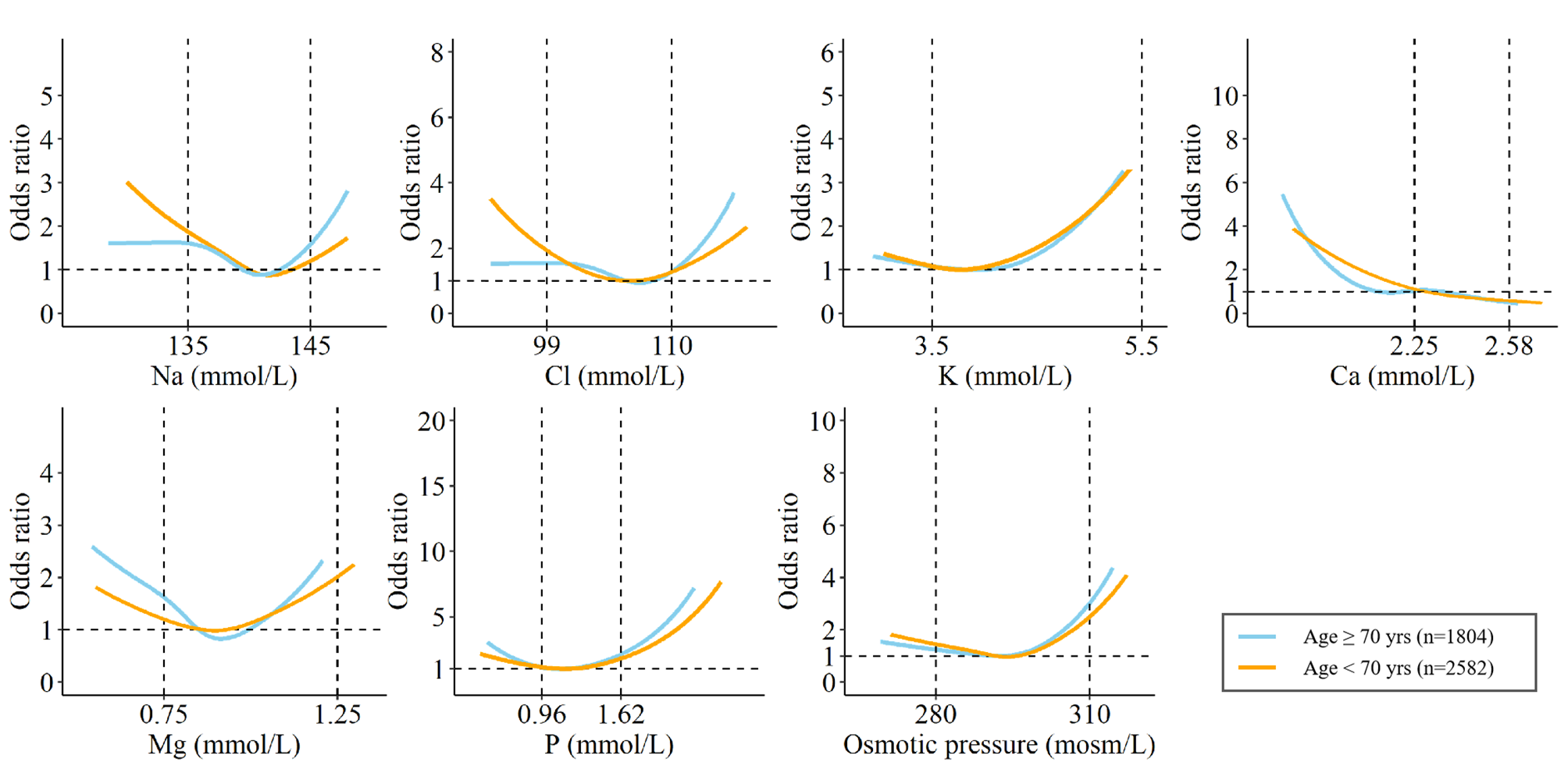

Supplement: Supplementary file 1 — Supplementary Information 1. [file 41598_2022_8597_MOESM1_ESM.tif]

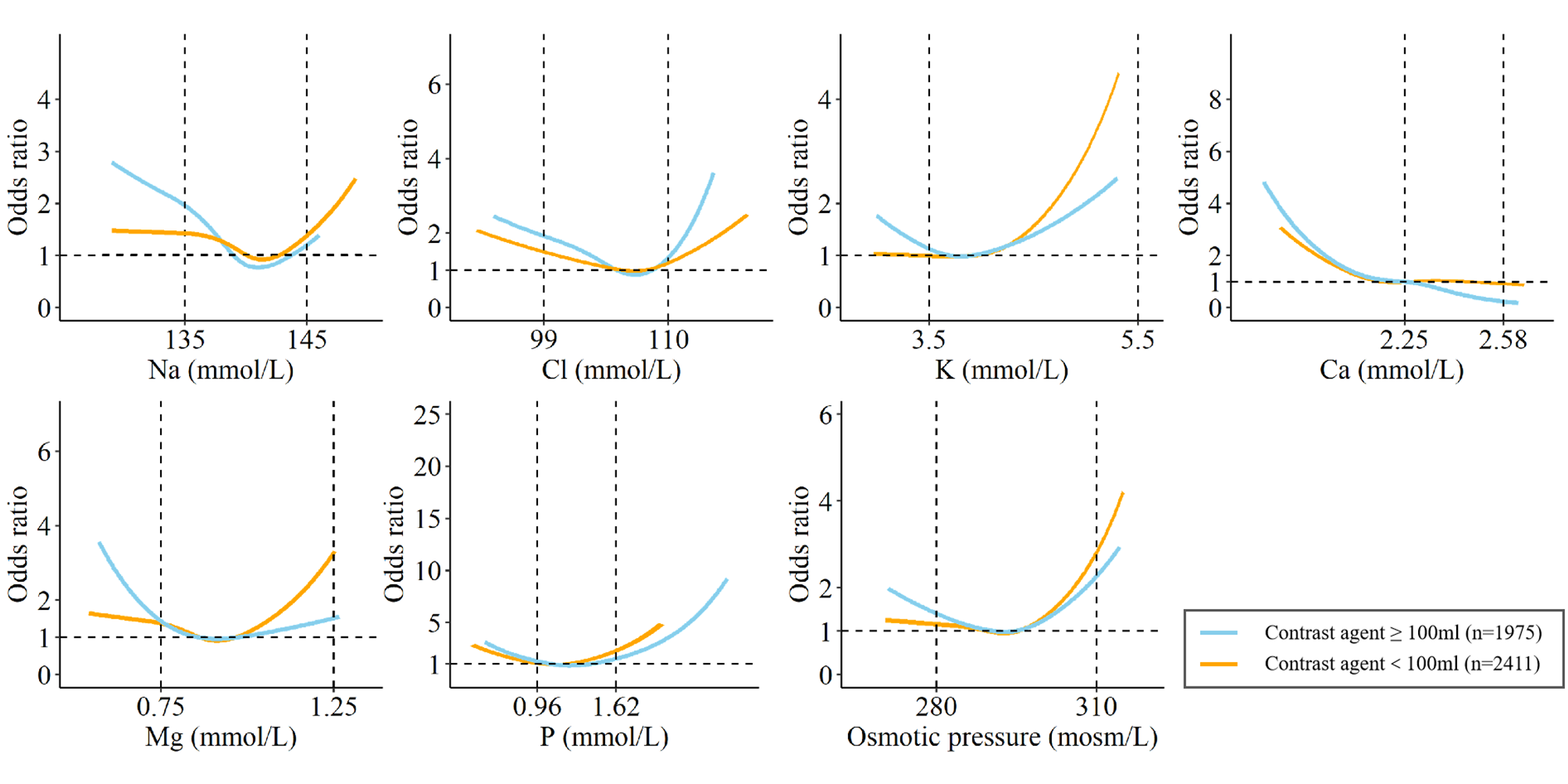

Supplement: Supplementary file 2 — Supplementary Information 2. [file 41598_2022_8597_MOESM2_ESM.tif]

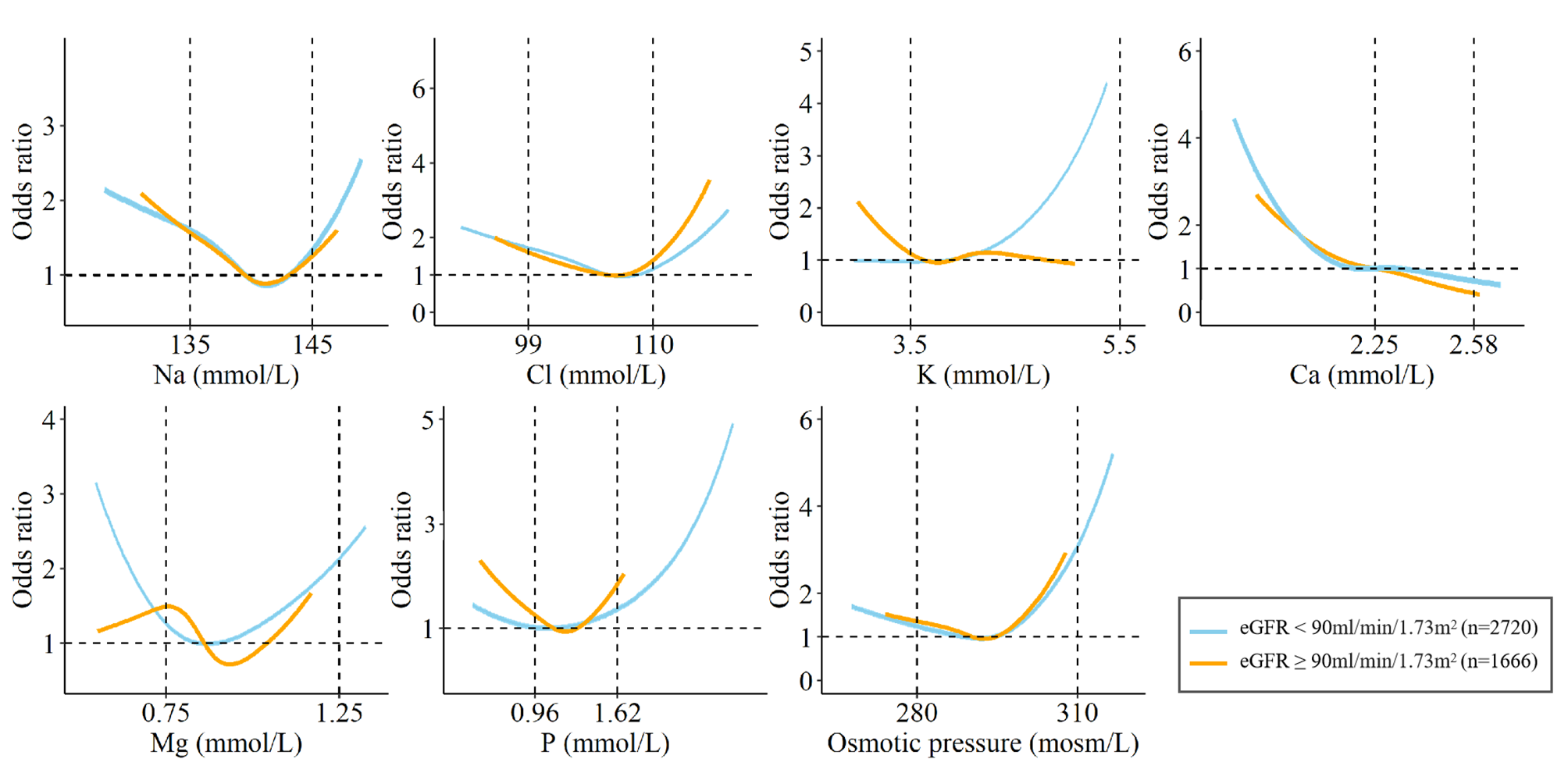

Supplement: Supplementary file 3 — Supplementary Information 3. [file 41598_2022_8597_MOESM3_ESM.tif]

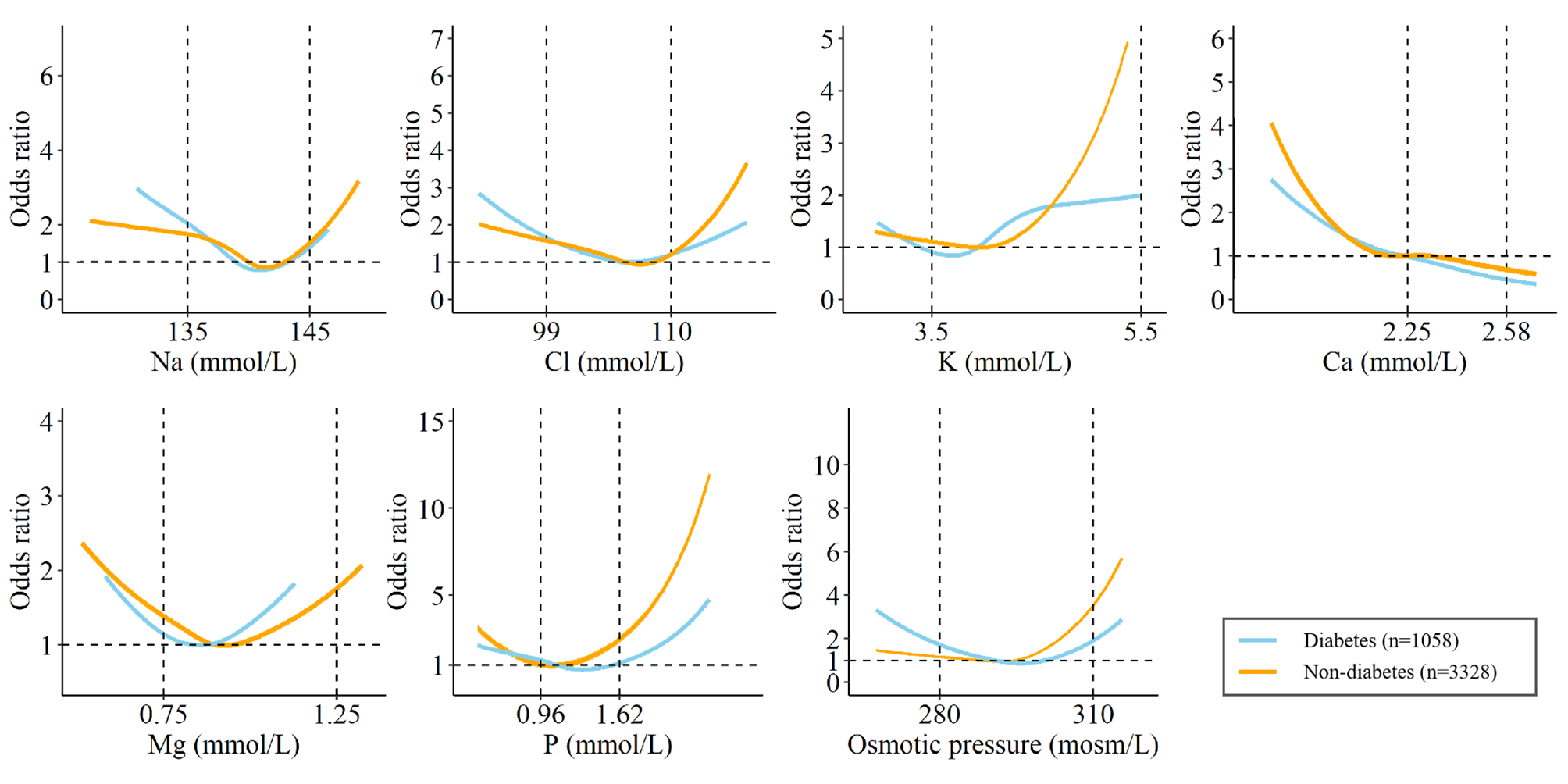

Supplement: Supplementary file 4 — Supplementary Information 4. [file 41598_2022_8597_MOESM4_ESM.tif]

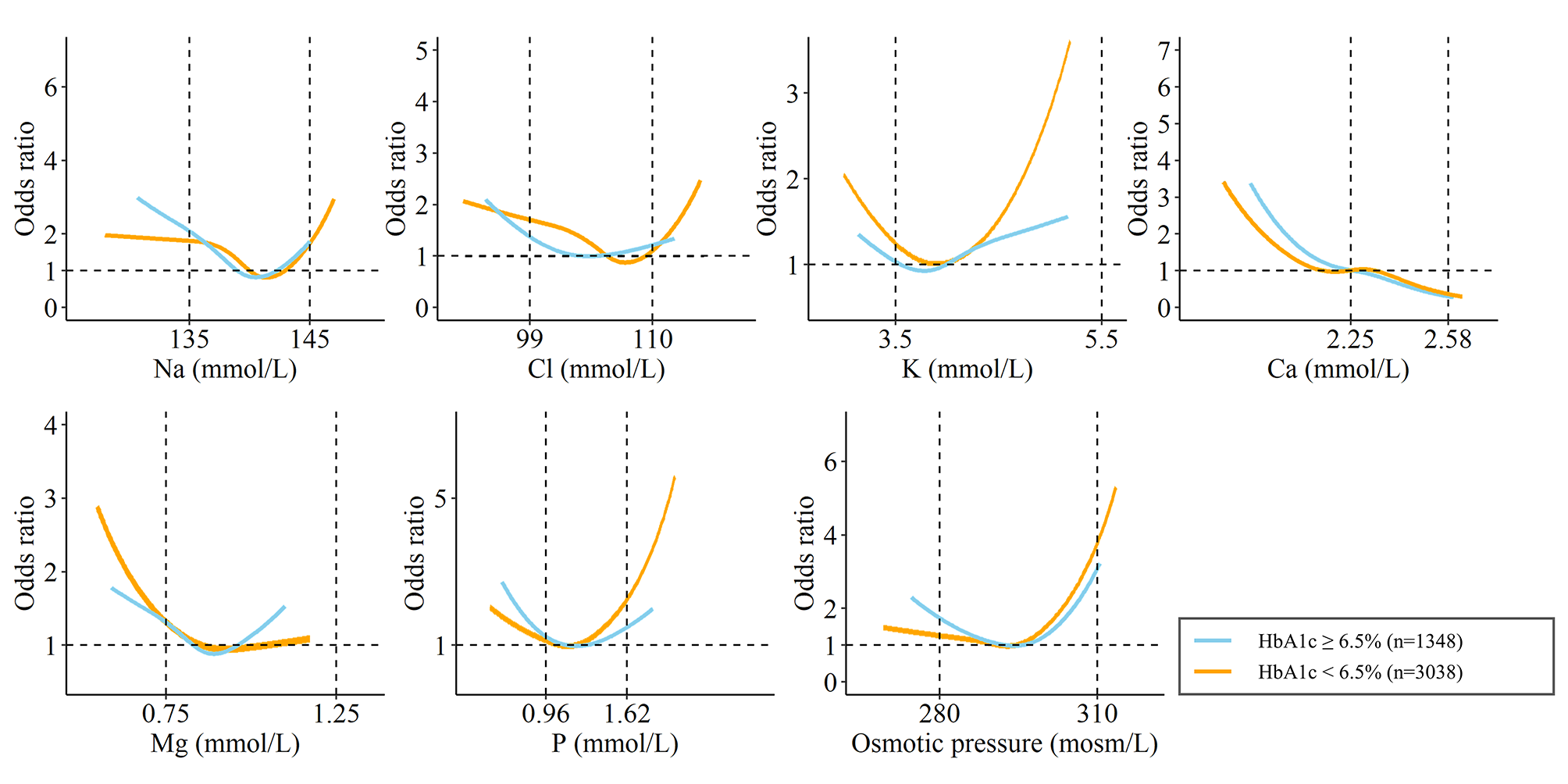

Supplement: Supplementary file 5 — Supplementary Information 5. [file 41598_2022_8597_MOESM5_ESM.tif]
