# Supplementary material for: Admission electrolyte and osmotic pressure levels are associated with the incidence of contrast-associated acute kidney injury
Source: Sci Rep. 2022 Mar 18;12:4714. doi: 10.1038/s41598-022-08597-z (PMC8933572; doi:10.1038/s41598-022-08597-z)
Supplement: Supplementary file 6 — Supplementary Information 6. [file 41598_2022_8597_MOESM6_ESM.docx]

**Supplemental Figure Legends**

**Supplemental Figure 1** Subgroup analysis of age on the CI-AKI incidence. All enrolled patients were divided into two subgroups according to the age of 70 years. The piecewise linear curve fitting by restricted cubic spline models of each subgroup (≥ 70 years or < 70 years) were presented for the association of sodium, chloride, potassium, calcium, magnesium, phosphate, and osmotic pressure distributions with CI-AKI incidence, respectively.

**Supplemental Figure 2** Subgroup analysis of contrast agent doses on the CI-AKI incidence. All enrolled patients were divided into two subgroups according to the doses of contrast agent. The piecewise linear curve fitting by restricted cubic spline models of each subgroup (Contrast agent ≥ 100 ml or Contrast agent < 100 ml) were presented for the association of sodium, chloride, potassium, calcium, magnesium, phosphate, and osmotic pressure distributions with CI-AKI incidence, respectively.

**Supplemental Figure 3** Subgroup analysis of eGFR levels on the CI-AKI incidence. All enrolled patients were divided into two subgroups according to the baseline levels of eGFR. The piecewise linear curve fitting by restricted cubic spline models of each subgroup (eGFR ≥ 90 ml/min/1.73m^2^ or eGFR < 90 ml/min/1.73m^2^) were presented for the association of sodium, chloride, potassium, calcium, magnesium, phosphate, and osmotic pressure distributions with CI-AKI incidence, respectively.

**Supplemental Figure 4** Subgroup analysis of the presence of diabetes mellitus on the CI-AKI incidence. All enrolled patients were divided into two subgroups according to the presence of diabetes mellitus. The piecewise linear curve fitting by restricted cubic spline models of each subgroup (Diabetes or Non-diabetes) were presented for the association of sodium, chloride, potassium, calcium, magnesium, phosphate, and osmotic pressure distributions with CI-AKI incidence, respectively.

**Supplemental Figure 5** Subgroup analysis of HbA1c levels on the CI-AKI incidence. All enrolled patients were divided into two subgroups according to the baseline levels of HbA1c. The piecewise linear curve fitting by restricted cubic spline models of each subgroup (HbA1c ≥ 6.5% or HbA1c < 6.5%) were presented for the association of sodium, chloride, potassium, calcium, magnesium, phosphate, and osmotic pressure distributions with CI-AKI incidence, respectively.
